# Which Factors Modulate Letter Position Coding in Pre-literate Children?

**DOI:** 10.3389/fpsyg.2021.708274

**Published:** 2021-08-05

**Authors:** María Fernández-López, Pablo Gómez, Manuel Perea

**Affiliations:** ^1^Department of Methodology of Behavioral Sciences and ERI-Lectura, Universitat de València, València, Spain; ^2^Department of Psychology, California State University, San Bernardino, Palm Desert, CA, United States; ^3^Center of Research in Cognition, Universidad Antonio de Nebrija, Madrid, Spain

**Keywords:** learning to read, orthographic processing, cognitive processing, pre-literate, transposed-letter effect

## Abstract

One of the central landmarks of learning to read is the emergence of orthographic processing (i.e., the encoding of letter identity and letter order): it constitutes the necessary link between the low-level stages of visual processing and the higher-level processing of words. Regarding the processing of letter position, many experiments have shown worse performance in various tasks for the transposed-letter pair judge-JUDGE than for the orthographic control jupte-JUDGE. Importantly, 4-y.o. pre-literate children also show letter transposition effects in a same-different task: TZ-ZT is more error-prone than TZ-PH. Here, we examined whether this effect with pre-literate children is related to the cognitive and linguistic skills required to learn to read. Specifically, we examined the relation of the transposed-letter in a same-different task with the scores of these children in phonological, alphabetic and metalinguistic awareness, linguistic skills, and basic cognitive processes. To that end, we used a standardized battery to assess the abilities related with early reading acquisition. Results showed that the size of the transposed-letter effect in pre-literate children was strongly associated with the sub-test on basic cognitive processes (i.e., memory and perception) but not with the other sub-tests. Importantly, identifying children who may need a pre-literacy intervention is crucial to minimize eventual reading difficulties. We discuss how this marker can be used as a tool to anticipate reading difficulties in beginning readers.

## Introduction

Whereas language is a unique and sophisticated human ability that emerges naturally in children, reading is a learned skill that needs intensive practice. In fact, reading acquisition is a complex process that involves functional brain changes and requires the correct execution of numerous mental functions (see Maurer et al., 2005). For this reason, children must have adequate perceptual and cognitive skills before the initial steps of reading instruction. Once acquired, reading becomes the most important tool for knowledge acquisition in academic settings and beyond.

In alphabetic scripts, readers can quickly map the visual input into abstract letter representations and, subsequently, into word representations (see Dehaene et al., 2005; Grainger et al., 2008). The emergence of these abstract letter representations would occur during the first 2 years of reading acquisition (Jackson and Coltheart, 2001). Consistent with this view, using Forster and Davis (1984) masked priming technique, Gomez and Perea (2020) found that, for Grade 2 readers, the identification time of a word like EDGE is virtually the same when rapidly preceded by the physically identical prime EDGE and when preceded by the nominally (but not physically) identical prime edge.

Importantly, the process of visual word recognition requires not only the encoding of the abstract identity of the letters that compose each word but also the encoding of the serial order of the words’ letters. If this process was absent, we would not be able to distinguish similarly spelled words like spot and stop. Notably, in a recent paper with adult readers, Schmitt and Lachmann (2020), demonstrated that when a target has to be identified in a string, processing occurs in serial order (i.e., from left to right) for letter stimuli, but not for strings composed of letters from an unknown alphabet (Cyrillic and Hebrew). At the same time, a considerable wealth of experiments with children and adults have shown that the encoding of letter order is only approximate: Readers often perceive jumbled words (e.g., JUGDE or CHOLOCATE) as the original words (see Perea and Lupker, 2003, 2004; Castles et al., 2007; Guerrera and Forster, 2008; Lupker et al., 2008). As serial order processing is a key component of a wide range of psychological processes, from perception to action (Logan, 2021), it is not surprising that the encoding of serial order is also an essential part of reading and literacy. The main goal of the present study is to shed some light on which cognitive factors are associated with pre-readers’ ability to encode letter position accurately.

In the context of reading development, Castles et al. (2007) proposed a “lexical tuning” model in which children encode progressively more precisely the letter positions within words. For instance, in a series of masked priming experiments, they found that the prime dark was much more effective at activating DARK in Grade 3 than in Grade 6 children. The rationale of this model is that, as reading abilities develop, letter position coding becomes more accurate (see Perfetti’s, 2017 lexical quality hypothesis, for a similar claim; but see Grainger and Ziegler, 2011, for a different view).^1^ Evidence supporting the lexical tuning model has been obtained not only with children of different ages but also with children of the same age: Better readers encode letter position more accurately than the worse readers (see Gómez et al., 2021; Pagán et al., 2021, for evidence with children and see also Andrews and Lo, 2012; Perea et al., 2016, for parallel evidence with adult readers). Furthermore, a poor encoding of serial order may lead to reading difficulties. Friedmann and Gvion (2001) were the first to report that some individuals present problems at encoding letter position, making frequent errors of letter migration within words—reading broad for board. This deficit, which has been termed “letter position” dyslexia, has been found in many different languages, including English (Kohnen et al., 2012; see Güven and Friedmann, 2019, for a recent review).

Somewhat surprisingly, examining how the encoding of letter order emerges in young readers and whether some preexisting abilities may help encode serial order in pre-readers has been overlooked in the literature. One of the few exceptions is the longitudinal experiment conducted by Duñabeitia et al. (2015). They used a same-different task with two sequentially presented four-letter strings, and children had to decide whether the letter strings were the same or different. The “different” trials were composed of pairs with two transposed letters (transposed-letter pairs; e.g., rzsk-rszk) and pairs with two replaced letters (replacement-letter pairs; e.g., rzsk-rhck). If letter position coding is flexible, transposed-letter pairs would be perceptually more similar than replacement-letter pairs, thus producing worse performance (e.g., more false positives). The “transposed-letter” effect is the difference in performance between these two conditions. Duñabeitia et al. (2015) tested the children three times: (1) in their year before preschool (*M =* 4.24 years) (2) in their preschool year (*M =* 5.21 years), and (3) in the first year of primary school (*M =* 6.32 years). They only found a transposed-letter effect when the children had learned to read (first-grade children; more error responses for transposed [42.9%] vs. replaced-letter pairs [30.6%]). Duñabeitia et al. (2015) concluded that “position uncertainty emerges as a consequence of literacy training” (p. 549).

**Figure 1:**
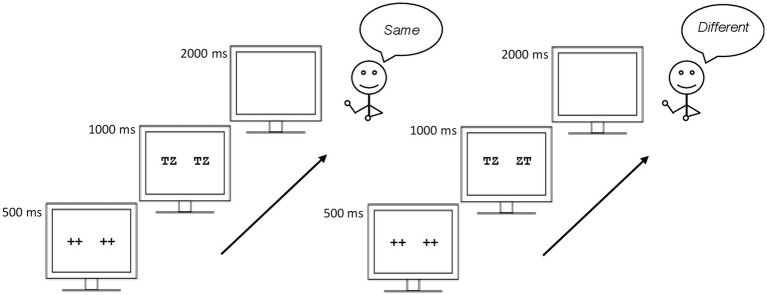
Depiction of the same-different task used in the Perea et al. (2016) study.

An interpretive issue in the Duñabeitia et al. (2015) experiment is that the pre-readers performed very poorly in the same-different task and the sensitivity index, *d*', was close to zero for both for replaced and transposed conditions (Perea et al., 2016). This pattern suggests that their version of the same-different task was too difficult for the pre-readers (i.e., the working memory load probably exceeded the children’s capacity; see Riggs et al., 2006); thus, one cannot make any inferences on these data. To draw firm conclusions on the encoding of the serial position of letters in pre-readers, Perea et al. (2016) simplified some elements of Duñabeitia et al.’s (2015) same-different task: (1) they used two-letter string pairs instead of four-letter string pairs (2) the pairs were presented simultaneously instead of sequentially, and (3) the responses were done verbally (i.e., saying “same” vs. “different”) instead of manually (pressing one of two buttons; see Figure 1). Along with “same” pairs (TZ-TZ), Perea et al. (2016) included the following “different” pairs: transposed-letter pairs (TZ-ZT), one-letter replacement pairs (TZ-PZ), and two-letter replacement pairs (TZ-PH). They found a sizeable transposed-letter effect in 4-years-old children (i.e., pre-readers). Specifically, the number of false positives (i.e., “same” responses) was greater to transposed-letter strings (TZ-ZT) than to 1 or 2 replacement-letter strings (TZ-PZ; TZ-PH). Perea et al. (2016) concluded that this pattern reflected a noisy perception of location order, common to all visual objects (see Gomez et al., 2008), rather than an effect that emerges with literacy. Notably, while not analyzed in their paper, shortly after conducting their experiment, Perea et al. (2016) collected the scores of these children in a battery of abilities related to early reading acquisition in Spanish (BIL battery; Sellés et al., 2008).

In the present study, we aim to take a step forward by exploring the potential precursors of letter position coding in pre-readers. To that end, we examined the relationship between the ability of pre-literate children to encode accurately the order of letters—taken from the Perea et al. (2016) experiment—with the five sub-tests related to reading readiness and subsequent reading success from the BIL battery: phonological and alphabetic awareness, metalinguistic knowledge, linguistic skills, and basic cognitive processes. The examination of this issue is important not only at a theoretical level but also at a practical level. Before learning to read, children must have acquired some perceptual, cognitive, and linguistic skills. Defining the early precursors of precise coding of letter position will shed light on the roots of the processing of serial order when reading letters in words. These analyses would allow us to identify children who may present some deficit (e.g., some mild forms of letter position dyslexia) and start intervening as soon as possible, preventing future reading difficulties.

Thus, in the present study, we examined the relationship between the sensitivity of the readers to distinguish transposed-letter pairs from identity pairs (e.g., TZ-ZT vs. TZ-TZ) and the scores of pre-readers (*M =* 4.5 years old) in phonological and alphabetic awareness, metalinguistic knowledge, linguistic skills, and basic cognitive processes (visual perception and sequential auditory memory) in the BIL battery (Sellés et al., 2008). We focused on transposed-letter pairs, as the mechanisms employed to discriminate TZ-ZT from ZT-ZT are based exclusively on letter order. The predictions are clear. In adult readers, basic cognitive processes, such as spatial and visual attention, have been assumed to play a key role in encoding letter position (see McCann et al., 1992; Gomez et al., 2008). If this generalizes to pre-readers, we expect a positive relationship between the abilities at discriminating TZ-ZT and the scores in these basic cognitive processes. This outcome would imply that educators could use this simple same-different task with a transposed-letter pairs to predict reading readiness before starting with the reading instruction. Furthermore, it may also operate as an incentive to design other tasks for pre-readers on perceptive and executive skills to prevent—or at least minimize—potential difficulties at locating letters within words during learning to read. In addition, we expect no relation between linguistic factors (i.e., phonological and alphabetic awareness, metalinguistic knowledge, and linguistic skills) and the sensitivity at distinguishing TZ-ZT in pre-readers—at the time of the experiment, the children did not know the consonant names.

## Materials and Methods

### Participants

They were the 20 preschoolers (*M =* 4.54 years; SD = 3.6; 7 girls) from a private school of Valencia (Spain). All of them were native speakers of Spanish with no learning developmental problems. An informed consent from their parents was obtained before running the experiment, and the study was approved by the Experimental Research Ethics Committee of the University of Valencia. At the time of testing, the preschoolers were starting to learn the vowels but they did not know the name or sound of the consonant letters (as confirmed by results of the BIL battery).

### Procedure

The experiment took place individually in a quiet room within the school premises. DMDX software (Forster and Forster, 2003) was employed for stimulus presentation and recording of the responses. A depiction of the procedure in the same-different task can be found in Figure 1. Accuracy was stressed in the instructions. Ten practice trials preceded the 64 experimental trials. Moreover, the children were assessed with a battery of abilities related to early reading acquisition in Spanish (BIL battery; Sellés et al., 2008).

### Materials

For the same-different task, the stimuli were 64 pairs of consonant strings made of two consonants. There were 16 trials in each of the conditions: (1) same pairs (TZ-TZ) (2) transposed-letter pairs (TZ-ZT) (3) one-letter replacement pairs (TZ-PZ), and (4) two-letter replacement pairs (TZ-PH). Four counterbalanced lists were created in a Latin square manner, so that each stimulus was rotated across the different conditions. The presentation of the items was randomized for each participant.

To assess the abilities related to early reading acquisition, we employed the BIL battery (Sellés et al., 2008). This battery comprises five sub-tests: phonological awareness, alphabetic awareness, metalinguistic knowledge, linguistic skills, and basic cognitive processes—we obtained a score from each sub-test. For the goals of the present study, we focused on the sub-test measuring basic cognitive processes. This sub-test explores a series of cognitive processes that take place when we face reading: (1) attention, which leads the mind to concentrate on specific stimuli; (2) sensation (i.e., detection and differentiation of sensory information); and (3) perception, which integrates sensory experiences and interprets them for recognition and identification (i.e., giving meaning to what has been selected and picked up at the attentional and sensory level), relying on the patterns stored in the (4) memory. To that end, the sub-test assesses the child’s sequential auditory memory and the ability to visually discriminate between similar letters and symbols (the child had to circle the symbols that were the same as a target; see Sellés et al., 2008, for a depiction of the other sub-tests).

## Results

To test whether better pre-reading skills (as measured by the BIL battery) were associated with better performance at differentiating between same and transposed-letter pairs in the same-different task, we conducted frequentist and Bayesian correlation analyses with JASP (Faulkenberry et al., 2020). To compute the Bayes factors, we used the default Cauchy distribution (centered around 0 and with a width parameter *δ* = 0.707; see Rouder et al., 2009; Wagenmakers et al., 2017, 2018, for discussion). Specifically, we examined the relation between *d*' (a measure of sensitivity obtained from the accuracy data of Perea et al., 2016) and the percentile scores in sub-tests of the BIL battery—of note, these findings were virtually the same if we had employed the raw scores from the sub-scales. For the computation of d’, we used the hit rate for same trials and the false alarm rate for the transposed-letter trials (TZ-TZ vs. TZ-ZT)—in signal detection theory, chance-level performance [d' = 0 or no sensitivity] occurs when the hit rate for the identical items is the equal to the false alarm rate for the different items. Of note, mean accuracy for same trials in the Perea et al. (2016) was 0.83; for different trials, it was 0.33 for transposed-letter strings and 0.68 and 0.88 for one-letter and two-letter replacement strings, respectively.

**Figure 2:**
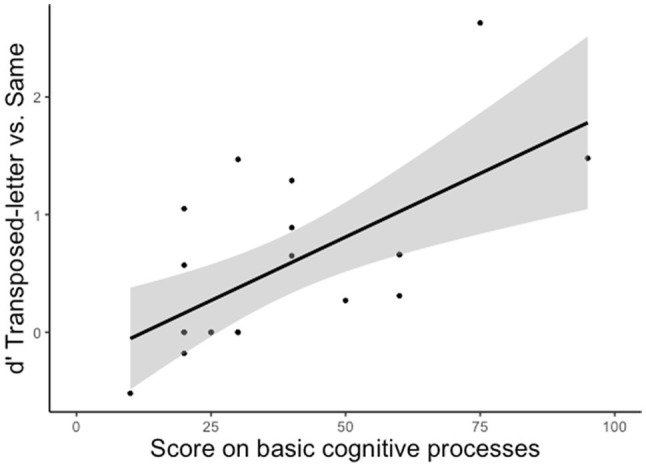
Correlation between the d' when discriminating transposed-letter pairs from same pairs and the scores on the basic cognitive processes of BIL.

Results of the correlational analyses in the present study showed that those children who better differ transposed letter from “same” pairs (TZ-ZT vs. TZ-TZ) had the higher scores in the sub-test on basic cognitive processes (*r =* 0.634, *p =* 0.003; see Figure 2). Indeed, the alternative hypothesis was 18.6 (BF_10_ = 18.559) times more likely than the null hypothesis with the present data (see Jeffreys, 1961, for interpretation of Bayes factors). In addition, there were no signs of a relationship between the children’s performance differentiating between same and transposed-letter pairs and the other (linguistic) sub-tests (all *ps >* 0.24, BFs10 < 0.528).

For completeness, we explored the relationship between performance in the replacement-letter conditions and the BIL battery; to this end, we computed separately d’s for same vs. one-letter replacement trials (TZ-TZ vs. TZ-PZ) and for same vs. two-letter replacement trials (TZ-TZ vs. TZ-PH), and then calculated the correlations between these two d’s and the sub-test of the BIL battery. None of these correlations produced evidence in favor of a relationship (all *p*s > 0.147; all BF_10_ < 0.478).

## Discussion

Identifying the cognitive precursors of reading is vital to determine those children who are ready to start learning to read and those who still need some cognitive maturation or some early intervention. This would prevent later reading difficulties and disorders and the frustration and psychological discomfort that such problems usually entail. With this matter in mind, in the present study, we scrutinized the roots of the mechanisms underlying the encoding of letter position in strings (i.e., one of the critical factors of efficient reading; see Castles et al., 2007; Logan, 2021). Specifically, we examined the relationship between the capability of pre-literates to differentiate between transposed-letter pairs and identity pairs (e.g., TZ-ZT vs. TZ-TZ) and these children’s scores in basic cognitive processes. Results showed that the pre-literate children who best differentiated between TZ-ZT and TZ-TZ in a same-different task were those with higher scores on basic cognitive processes (see Figure 2). Notably, the sub-test of basic cognitive processes was not generically associated with sensitivity in the same-different task (i.e., it was not related to performance for replacement-letter trials); instead, it is uniquely associated with accuracy in letter position coding. Thus, at the theoretical level, this outcome reflects that basic cognitive skills shape the ability to encode serial order in letter strings (e.g., a smaller value of the parameter responsible for perceptual uncertainty in models of letter position coding; see Gomez et al., 2008; Davis, 2010). Furthermore, at an applied/educational level, our findings imply that a simple same-different task can be used to assess reading readiness: the better the performance in this task, the better the encoding of letter order, diminishing the chances of letter position dyslexia.

In addition, our findings suggest that the preparing-to-reading arises early in development with some non-specialized processes that would be recruited and adjusted to guide the subsequent functional reading progress (see Lachmann and van Leeuwen, 2014). Further support to this idea can be found in the study of Saygin et al. (2016). They found that the cortical location of the visual word form area (i.e., the brain region specialized for letter string; Dehaene and Cohen, 2011) at age 8 (when children read) can be predicted by the distinctive connectivity of the same region at age 5 (pre-literates). Taken together, these studies emphasize that early detection of deficiencies in the visual analysis of the input is crucial to prevent later reading difficulties (Friedmann and Gvion, 2001; Shetreet and Friedmann, 2011). This is consistent with the assumption that children with reading difficulties have a general impairment in domains other than linguistic (e.g., an impairment in multisensory integration; see Gori and Facoetti, 2014; Lachmann and van Leeuwen, 2014). Therefore, there are possibly many (complementary) ways to test whether pre-readers are prepared to starting reading learning (e.g., the same-different task or the “avatar task”; see Perea et al., 2014); this would be a valuable endeavor for the future studies.

We acknowledge that the present study comes with some limitations. Firstly, because of the correction criteria of the BIL test, it was not possible to obtain separate scores for the tasks that make up the basic cognitive processes sub-test (sequential auditory memory and visual discrimination). Furthermore, although the processes assessed in the basic cognitive sub-test were not linguistic in nature, the stimuli contained symbols, letters, and words, thus, making it difficult to clearly disentangle basic cognitive processes and verbal processes. Future tests should be more specific to characterize all possible aspects that shape the cognitive processes of pre-literates. In addition, it would have been desirable to have obtained further data from the same children once they started reading learning. These data would have allowed us to test whether the findings in the same-different task with transposed letters in pre-literate children were a good predictor of letter position coding once children acquired knowledge about letters. Furthermore, these longitudinal data would have also allowed us to examine the interplay between the emergence of orthographic processing during learning and the scores in cognitive and linguistic processes. Indeed, once the children start to read, other elements would begin playing a significant role, such as alphabetic knowledge or phonologic awareness (see Dehaene et al., 2015).

A complementary strategy for the future research would be to run parallel longitudinal same-different experiments on serial order using to-be-learned letters vs. unknown letters (e.g., letters from another alphabet). The data pattern should be similar for the pre-readers for both types of stimuli, but one would expect differences when the children learn to read. Critically, these differences could be considered as markers of the emergence of orthographic processing (see Grainger, 2018). While this approach is ideal on an *a priori* basis, it suffers from various methodological issues. One would need to design a feasible task for children of different tasks that minimizes both ground and ceiling effects. However, it is challenging to create a task achievable for pre-literates and complicated enough to draw differences among developing readers. For instance, deciding whether two four-letter strings are the same is extremely challenging for pre-literates, whereas deciding whether two-letter strings are the same may be too easy for developing readers (see Perea et al., 2016, for discussion). As a result, it is very difficult to experimentally study the emergence and development of orthographic processes in pre-readers. To further complicate matters, there are also other potential limitations, such as the lack of control for prior letter knowledge and other linguistic elements in pre-readers, or that the duration of experiments for pre-readers would need to be quite short to keep them attentive. An alternative is to design laboratory analogs of children’s reading acquisition that consists of training adults to read a novel script (see Fernández-López et al., 2021; see also Chetail, 2017; Taylor et al., 2017). This approximation is not as ecological as one would desire (see Maurer et al., 2010; Taylor et al., 2017), but it definitively increases the control on the process of acquiring the novel orthography.

In sum, the early identification of potential problems that may slow down reading development is of fundamental importance for psychologists and educators. In the present study, we found that those pre-readers who performed better in basic cognitive processes tended to be those who would encode more accurately letter position in a simple same-different task. This finding highlights that learning to read should not be based solely on letter knowledge and phonological decoding. We also need to consider that learning to read is built on a basic cognitive foundation, probably related to multisensory integration based on visual attention.

## Data Availability Statement

Publicly available datasets were analyzed in this study. This data can be found at: https://osf.io/hz7m2/?view_only=2073b5df420e4d5f95c627ec4ee6e81b.

## Ethics Statement

The studies involving human participants were reviewed and approved by Comité de Ética de Investigación en Humanos (CEIH). Written informed consent to participate in this study was provided by the participants’ legal guardian/next of kin.

## Author Contributions

MP, PG and MF-L contributed to conception, design of the study, and performed the statistical analysis. PG organized the database. MF-L wrote the first draft of the manuscript. MF-L and MP wrote sections of the manuscript. All authors contributed to manuscript revision, read, and approved the submitted version.

## Conflict of Interest

The authors declare that the research was conducted in the absence of any commercial or financial relationships that could be construed as a potential conflict of interest.

## Publisher’s Note

All claims expressed in this article are solely those of the authors and do not necessarily represent those of their affiliated organizations, or those of the publisher, the editors and the reviewers. Any product that may be evaluated in this article, or claim that may be made by its manufacturer, is not guaranteed or endorsed by the publisher.

## Footnotes

^1^In fairness to Grainger and Ziegler (2011), their dual-route model of visual word recognition focuses on the initial steps of learning to read. The serial letter encoding, which involves precise letter position encoding, would emerge first in reading development (phonological route). Later in development, the parallel encoding of the word’s letters (orthographic route) would make letter position coding coarser.
